# Supplementary material for: Disinfection Strategies for Carbapenem-Resistant Klebsiella pneumoniae in a Healthcare Facility
Source: Antibiotics (Basel). 2022 May 30;11(6):736. doi: 10.3390/antibiotics11060736 (PMC9219816; doi:10.3390/antibiotics11060736)
Supplement: Supplementary file 1 [file antibiotics-11-00736-s001.zip › antibiotics-1722664-supplementary-done.pdf]

antibiotics

## Supplementary Materials

### 1. Figures

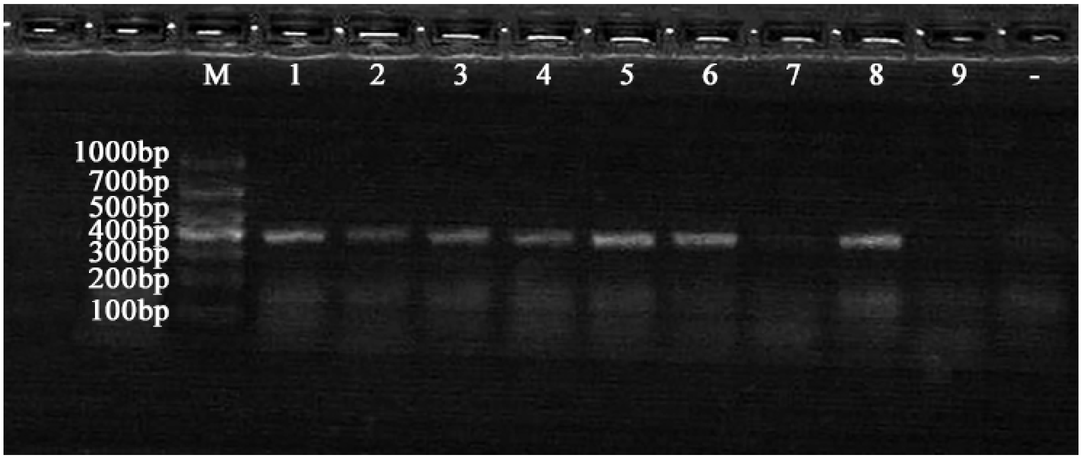


**Figure S1.** Agarose gel electrophoresis image of *oqxA* gene. "M" is 1000bp DNA Marker. "1, 2, 3, 4, 5, 6, 7, 8" are positive specimens. "9" is negative specimen. "-" is negative control.

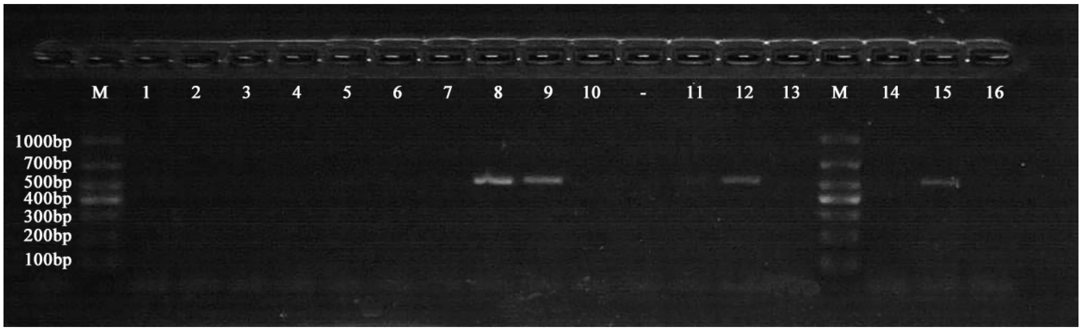


**Figure S2.** Agarose gel electrophoresis image of *oqxB* gene. "M" is 1000bpDNA Marker. "8, 9, 12, 15" are positive specimens. "1, 2, 3, 4, 5, 6, 7, 10, 11, 13, 14, 16" are negative specimens. "-" is negative control.

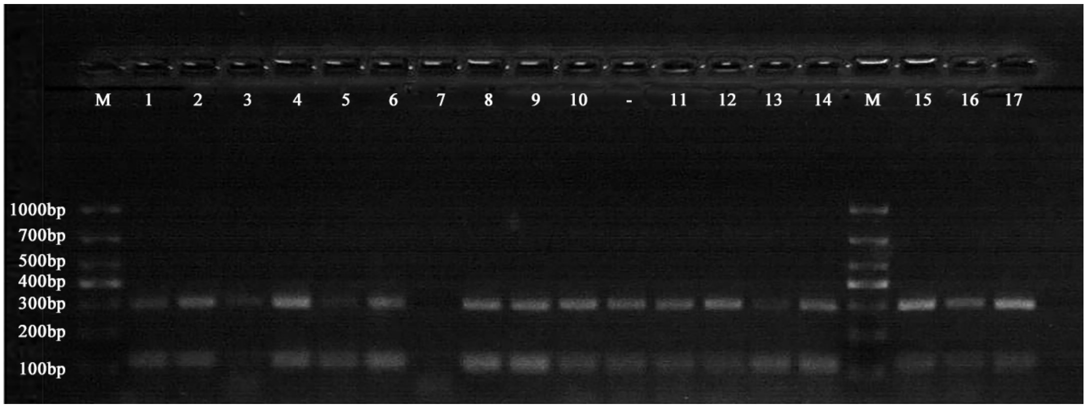


**Figure S3.** Agarose gel electrophoresis image of *qacE△1-sul1* gene. "M" is 1000bpDNA Marker. "1, 2, 3, 4, 5, 6, 8, 9, 10, 11, 12, 13, 14, 15, 16, 17" are positive specimens. "7" is negative specimens. "-" is negative control.

## 2. Tables

**Table S1.** Primer sequence of efflux pump genes.

| Efflux pump family | Gene | Primer Sequence (5′→3′) | Size (bp) |
|---|---|---|---|
| RND family | *oqxA*[22] | P1:CTCGGCGCGATGATGCT<br>P2:CCACTCTTCACGGGAGACGA | 392 |
| | *oqxB*[22] | P1:TTCTCCCCCGGCGGGAAGTAC<br>P2:CTCGGCCATTTTGGCGCGTA | 512 |
| SMR family | *qacE△1-sul1*[21] | P1:AGCGAGGGCTTTACTAAGC<br>P2:ATTCAGAATGCCGAACACCG | 300 |

*Note: RND, resistance-nodulation cell division. SMR, small multidrug resistance.*

**Table S2.** Departmental distribution of isolated CRKP strains from 2015 to 2019.

| | 2015 | 2016 | 2017 | 2018 | 2019 | Total |
|---|---|---|---|---|---|---|
| ICU | 2 | 1 | 18 | 18 | 28 | 67 |
| Neurology/Neurosurgery | 0 | 3 | 4 | 5 | 5 | 17 |
| Rehabilitation | 0 | 1 | 3 | 3 | 6 | 13 |
| Hepatopancreatobiliary | 1 | 1 | 3 | 3 | 2 | 10 |
| Hematology | 1 | 1 | 1 | 4 | 4 | 11 |
| Urology/Nephrology | 1 | 0 | 6 | 3 | 1 | 11 |
| Orthopaedics | 0 | 1 | 2 | 0 | 1 | 4 |
| Respiratory | 0 | 1 | 0 | 0 | 3 | 4 |
| Emergency | 0 | 1 | 2 | 1 | 3 | 7 |
| Endocrinology | 0 | 1 | 2 | 1 | 0 | 4 |
| Dermatology | 0 | 1 | 0 | 0 | 0 | 1 |
| Stomatological department | 0 | 0 | 1 | 0 | 0 | 1 |
| Reproductive Center | 0 | 0 | 1 | 0 | 0 | 1 |
| Gastroenterology | 0 | 0 | 2 | 2 | 0 | 4 |
| Cardiovascular surgery | 0 | 0 | 0 | 1 | 0 | 1 |
| Neonatology | 0 | 0 | 0 | 1 | 1 | 2 |
| Oncology | 0 | 0 | 0 | 2 | 1 | 3 |

| | | | | | | |
|---|---|---|---|---|---|---|
| Chinese Internal Medicine | 0 | 0 | 0 | 0 | 1 | 1 |
| Total | 5 | 12 | 45 | 44 | 56 | 162 |

*Note: The sudden increase in the detection amount in 2017 mainly occurred in the ICU department; ICU, intensive care unit.*

**Table S3.** The distribution of specimen types in the departments with CRKP detection

| | ICU | Neurology/Neurosurgery | Rehabilitation | Urology/Nephrology | Hematology | Hepatopancreatobiliary |
|---|---|---|---|---|---|---|
| Sputum | 37 | 11 | 10 | 0 | 1 | 2 |
| Ascites/Abdominal drainage fluid | 11 | 0 | 0 | 1 | 0 | 4 |
| Blood | 5 | 2 | 0 | 0 | 5 | 0 |
| Catheter | 3 | 0 | 0 | 0 | 2 | 0 |
| Urine | 2 | 1 | 3 | 9 | 0 | 0 |
| Wood secretion | 2 | 2 | 0 | 1 | 0 | 0 |
| Broncho-alveolar lavage | 2 | 0 | 0 | 0 | 0 | 0 |
| Stool | 0 | 0 | 0 | 0 | 3 | 0 |
| Puncture fluid | 1 | 1 | 0 | 0 | 0 | 0 |

| | | | | | | |
|---|---|---|---|---|---|---|
| Bile | 1 | 0 | 0 | 0 | 0 | 4 |
| Pelvic drainage fluid | 1 | 0 | 0 | 0 | 0 | 0 |
| Pleural drainage fluid | 1 | 0 | 0 | 0 | 0 | 0 |
| Vagina secretion | 1 | 0 | 0 | 0 | 0 | 0 |
| Total | 67 | 17 | 13 | 11 | 11 | 10 |

*Note: ICU, intensive care unit.*
